# The Muscle Oxidative Regulatory Response to Acute Exercise Is Not Impaired in Less Advanced COPD Despite a Decreased Oxidative Phenotype

**DOI:** 10.1371/journal.pone.0090150

**Published:** 2014-02-28

**Authors:** Ilse G. M. Slot, Bram van den Borst, Valéry A. C. V. Hellwig, Esther Barreiro, Annemie M. W. J. Schols, Harry R. Gosker

**Affiliations:** 1 Department of Respiratory Medicine, NUTRIM School for Nutrition, Toxicology and Metabolism, Maastricht University Medical Center+, Maastricht, The Netherlands; 2 Pulmonology Department-Muscle and Respiratory System Research Unit (URMAR), Research Institute of Hospital del Mar (IMIM), Department of Experimental and Health Sciences (CEXS), Pompeu Fabra University (UPF), Barcelona Biomedical Research Park (PRBB), Barcelona, Spain; 3 Network of Excellence in Respiratory Research (CIBERES), Institute of Health Carlos III (ISCIII), Bunyola, Majorca, Balearic Islands, Spain; Southern Illinois University School of Medicine, United States of America

## Abstract

Already in an early disease stage, patients with chronic obstructive pulmonary disease (COPD) are confronted with impaired skeletal muscle function and physical performance due to a loss of oxidative type I muscle fibers and oxidative capacity (i.e. oxidative phenotype; Oxphen). Physical activity is a well-known stimulus of muscle Oxphen and crucial for its maintenance. We hypothesized that a blunted response of Oxphen genes to an acute bout of exercise could contribute to decreased Oxphen in COPD. For this, 28 patients with less advanced COPD (age 65±7 yrs, FEV_1_ 59±16% predicted) and 15 age- and gender-matched healthy controls performed an incremental cycle ergometry test. The Oxphen response to exercise was determined by the measurement of gene expression levels of Oxphen markers in pre and 4h-post exercise quadriceps biopsies. Because exercise-induced hypoxia and oxidative stress may interfere with Oxphen response, oxygen saturation and oxidative stress markers were assessed as well. Regardless of oxygen desaturation and absolute exercise intensities, the Oxphen regulatory response to exercise was comparable between COPD patients and controls with no evidence of increased oxidative stress. In conclusion, the muscle Oxphen regulatory response to acute exercise is not blunted in less advanced COPD, regardless of exercise-induced hypoxia. Hence, this study provides further rationale for incorporation of exercise training as integrated part of disease management to prevent or slow down loss of muscle Oxphen and related functional impairment in COPD.

## Introduction

Loss of skeletal muscle oxidative phenotype (Oxphen) is prevalent in chronic obstructive pulmonary disease (COPD) [1]-[3]. It includes a proportional shift from slow-oxidative type I muscle fibers towards the fast-glycolytic type II fibers, associated with a reduced capacity of oxidative metabolism and in advanced disease also mitochondrial dysfunction [4]. Loss of muscle Oxphen is related to functional impairments, such as a reduction in muscle endurance [4], whole body exercise capacity, and mechanical efficiency [5], and it has been proposed as a driver of cardiovascular and metabolic risk [6] and cachexia [7]. Loss of muscle Oxphen is most prominent in severe COPD, but we recently showed that the process is already ongoing in patients with less advanced COPD [8]. Hence, this earlier stage of the disease is of particular importance to gain mechanistic insight into the onset of Oxphen loss in COPD.

Exercise is an important stimulus for maintenance of muscle Oxphen. Because patients with COPD are physically less active [9], it is tempting to attribute Oxphen loss in these patients to disuse. However, several recent reports could not show such an association between physical activity level and Oxphen [8], [10], [11]. Physiologically, muscle Oxphen is progressively stimulated and maintained by repeated bouts of exercise through induction of among others peroxisome proliferator-activated receptor (PPAR) coactivator-1α (PGC-1α), PPAR-α and -δ, and mitochondrial transcription factor A (TFAM) [12], [13]. We hypothesized that the response of Oxphen regulation to acute bouts of exercise is blunted in COPD, which could contribute to a loss of muscle Oxphen irrespective of physical activity level. To test this hypothesis we compared Oxphen markers and their regulators in muscle biopsies obtained before and after an acute bout of exercise, between less advanced COPD patients and healthy controls who were matched for age and body mass index (BMI).

Hypoxia is known to increase muscle glycolytic capacity relative to oxidative capacity [14], [15]. In humans, hypoxic exposure also induces loss of muscle oxidative capacity by reducing mitochondrial volume (reviewed in [16]). Exercise-induced oxygen desaturation even occurs in some patients with less advanced COPD, potentially reflecting or leading to hypoxia in the exercising muscle. Experimental research in mice has shown that exposure to acute hypoxia can also induce oxidative stress in skeletal muscle mitochondria [17]. Indeed, elevated levels of oxidative stress markers have been shown in muscles of COPD patients with chronic hypoxemia [18]. Moreover, enhanced exercise-induced oxidative stress has frequently been observed in muscle of patients with severe COPD [19], particularly in the wasted subgroup [20]. Interestingly, in severe patients, oxidative stress is inversely associated with muscle endurance [21] and *in vitro* permeabilized muscle fibers of patients with severe COPD produce significantly more reactive oxygen species than those of healthy controls [22]. This is possibly related to a fiber-type shift, since studies in rats have shown that mitochondria within type II fibers release significantly more H_2_O_2_ than those within oxidative fibers [23]. As such, oxygen desaturation and/or muscle oxidative stress could mediate an anticipated blunted Oxphen response in COPD. A secondary objective was therefore to determine the involvement of exercise-induced oxygen desaturation and oxidative stress in the response to an acute exercise bout.

## Methods

### Ethics statement

Written informed consent was obtained from all subjects and the ethical review board of the Maastricht University Medical Center+ approved the study (08-2-059). The study was registered at www.trialregister.nl as NTR1402.

### Patient characteristics

The study population included 28 clinically stable mild to moderate COPD patients (FEV_1_ 59±16% predicted; *P*_a_,O_2_ 9.3±1.0 kPa) and 15 healthy controls. Recruitment of patients and controls and exclusion criteria were previously described [8]. Briefly, patients were recruited from the outpatient clinic of Maastricht University Medical Center+ (MUMC+, Maastricht, The Netherlands) and via advertisements in local newspapers. Exclusion criteria were long-term oxygen therapy, oral corticosteroid use, acute exacerbation requiring hospital admission in the past eight weeks, rehabilitation in the past six months and known co-morbidities potentially interfering with study outcome parameters. Healthy controls were recruited via advertising in local newspapers and absence of co-morbidities and airflow limitation was verified through history-taking by a physician, and pulmonary function tests. Healthy controls were matched to COPD patients on age, BMI and sex distribution. Study subjects were carefully characterized regarding body composition, lung function, smoking status and daily physical activity level, as reported previously [8]. The protocol used to determine quadriceps function on a dynamometer was previously described [8]. Briefly, under strong encouragement, subjects performed thirty sequential volitional maximal contractions at an angular velocity of 90°/s. Isokinetic quadriceps endurance was determined as proportional decline in peak torques (relative to the highest peak torque) per repetition (%/rep).

### Experimental protocol

To study the response of Oxphen regulators and oxidative stress to acute exercise, subjects performed an incremental load cycle ergometry test. Quadriceps muscle biopsies were obtained directly before and 4 h after the exercise test for Oxphen and oxidative stress analyses to obtain a maximal response of metabolic and oxidative stress-related genes after exercise. During the entire exercise test, oxygen saturation was measured by means of traditional finger pulse oximetry, as well as using a portable continuous-wave near-infrared spectroscopy (NIRS) system to examine oxygen saturation locally at the muscular level. Arterial punctures were performed before exercise and at maximal exertion for gas and lactate analyses.

### Acute exercise protocol

All subjects performed an incremental load cycle ergometry test. The rationale for this protocol was to invoke a rapid exercise response in terms of Oxphen regulation particularly in the quadriceps muscle (see below). Peak oxygen uptake (*V*′o_2peak_) and peak work load were determined as previously described [24]. Anaerobic threshold was determined from ergospirometry data using the v-slope method [25]. Arterial punctures of the radial artery at rest and at *V*′o_2peak_ were available from *n* = 19 COPD patients and *n* = 11 healthy subjects. Arterial blood gas and lactate concentrations were analyzed on a Chiron blood gas analyzer 865 (Chiron Diagnostics, Emeryville, CA).

### Arterial and peripheral muscle oxygenation

Arterial oxygen saturation was continuously monitored using a finger pulse oximeter. By *post hoc* stratification the patients were divided into those who desaturated during exercise (*S*_p_,o_2_ fall ≥ 4%; COPD_D_, *n* = 13) and those who did not (*S*_p_,o_2_ fall<4%; COPD_ND_, *n* = 15) according to ATS criteria [26]. Quadriceps muscle oxygenation was measured using the PortaMon portable continuous-wave near-infrared spectrophotometer (NIRS, Artinis Medical Systems, Zetten, The Netherlands). The probe was attached with Velcro straps on the *vastus lateralis* of the non-biopsied leg approximately 10 cm proximally from the knee joint, and covered with a black cloth to eliminate environmental light. Measurements started 3 minutes before exercise while the subject was still at rest and continued for 2–5 minutes after the exercise test until the signal had stabilized. Baseline values were established during 3 minutes of unloaded cycling before the incremental exercise test. Tissue oxygenation was measured continuously. Traces were analyzed with OxySoft Software (v2.1.2; Artinis Medical Systems). The tissue saturation index (TSI) was calculated using spatial resolved spectroscopy using the equation below, with [O_2_Hb] and [HHb] representing concentrations of oxygenated and deoxygenated hemoglobin/myoglobin.
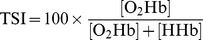



Values were reported as change from baseline (Δ). Using mono-exponential curve fitting, the time constant of muscle reoxygenation (tau, τ) was determined from the slope of the TSI curve during the reoxygenation phase. Twelve subjects (4 COPD_ND_, 4 COPD_D_ patients, and 4 healthy controls) were excluded from further NIRS analyses mainly because their skin fold at the site of the NIRS measurement was greater than 3 cm (which is the operational limit for the NIRS probe), or because the signal was poor or did not show a clear drop in TSI.

### Muscle biopsy and analyses

Biopsies were obtained at rest and 4 h after the exercise test from quadriceps muscle (*vastus lateralis*) of the dominant leg from two separate incisions approximately 2 cm apart using the needle biopsy technique [27]. The interval between the exercise test and the second biopsy was chosen after careful study of the literature to obtain a maximal response of metabolic genes after exercise (e.g., [28], [29]). Muscle tissue was frozen in melting isopentane precooled in liquid nitrogen and stored in aliquots at −80°C for histology and molecular analyses.

Techniques for sample preparation for analyses of fiber-type composition, gene expression, oxidative stress markers, metabolic enzyme activity as well as the corresponding analyses have been previously described [8]. Briefly, fiber-type composition was determined on 5 µm serial cryosections using antibodies against myosin heavy chain (MyHC) I, MyHC IIa (both from Developmental Studies Hybridoma Bank, University of Iowa, USA), and laminin (Sigma, Zwijndrecht, the Netherlands). Fiber-type classification was aided by myosin ATPase-activity staining with acidic pre-incubation at pH 4.40 [30]. For qRT-PCR, RNA was extracted from 10–30 mg muscle tissue using the ToTALLY RNA™ Kit (Ambion Ltd., Foster City, CA, USA) according to the supplier's protocol, followed by genomic DNA removal with the RNeasy Mini Kit with RNase-free DNase (Qiagen, Venlo, The Netherlands). 400 ng RNA was reverse transcribed to cDNA with anchored oligo(dT) primers according to the supplier's protocol (Transcriptor First Strand cDNA Synthesis kit, Roche Diagnostics, Woerden, The Netherlands). qRT-PCR primers were designed based on Ensembl transcript sequences or selected from literature [31], [32] and ordered from Sigma Genosys (Zwijndrecht, the Netherlands), with primer details shown in Table S1 and Table S2 in File S1. Using these analyses, regulators and markers of oxidative and glycolytic metabolism as well as oxidative stress were assessed. Contents of antioxidant enzymes were determined with the western blot protocol using anti-catalase antibody (Calbiochem, San Diego, CA, USA) and anti-manganese superoxide dismutase and anti-Cu/Zn superoxide dismutase antibodies (Santa Cruz Biotechnology, CA, USA). Protein content of oxidative stress markers were identified using antibodies for protein carbonylation (anti-2,4-DNP moiety antibody, Oxyblot kit, Chemicon International Inc., Temecula, CA, USA), total protein nitration (anti-3-nitrotyrosine antibody, Invitrogen, Eugene, OR, USA), and total malondialdehyde (MDA)-protein adducts (anti-MDA antibody, Academy Biomedical Company Inc., Houston, TX, USA). For protein carbonylation, carbonyl groups in the protein side chains were first derivatized to 2,4-dinitrophenylhydrazone (DNP) using the Oxyblot kit (Chemicon International Inc., Temecula, CA, USA) according to the manufacturer's instructions. Exercise responses of gene expression and oxidative stress product variables were summarized with fold inductions, which were calculated by dividing post-exercise values by baseline values. Contents of representative subunits of oxidative phosphorylation (OXPHOS) complexes were determined by western blot using an anti-total OXPHOS antibody cocktail (MS604-300, Abcam, Cambridge, UK) with GAPDH (anti-GAPDH, #2118, Cell Signaling Technology, Leiden, The Netherlands) as a loading control. Metabolic activities for citrate synthase (CS, EC 2.3.3.1) and 3-hydroxyacyl-CoA dehydrogenase (HADH, EC 1.1.1.35) were assayed spectrophotometrically (Multiskan Spectrum; Thermo Labsystems, Breda, The Netherlands) as previously described [33]-[35]. Absolute CS and HADH activities were normalized to total protein.

### Statistics

The assumptions of normality and homogeneity of variances were checked for all experimental groups with the Shapiro-Wilk test and Levene's test. Three different sets of statistical comparisons were made. First, baseline comparisons between the COPD patients and controls were tested using independent Student's *t* test or Mann-Whitney test as appropriate. Comparisons between COPD_ND_, COPD_D_ and control groups were made using one-way ANOVA (*post hoc* Gabriel), Welch's ANOVA (*post hoc* Games-Howell) or Kruskal-Wallis non-parametric ANOVA (*post hoc* Mann-Whitney U with Bonferroni correction) as appropriate. Discontinuous variables were tested using Fisher's Exact test. These same tests were subsequently employed to analyze differences in exercise-induced response of skeletal muscle metabolic genes and oxidative stress markers *between* the groups (expressed as fold inductions), whereas paired Student's *t* test or Wilcoxon signed-rank test were performed to analyze such changes *within* each study group. Correlations were tested using Pearson's correlation coefficient, or Spearman's ρ in case of non-normally distributed data. Analyses were performed using IBM SPSS Statistics 20.0 (IBM Corp., Armonk, NY). A *p*-value<0.05 was considered statistically significant.

## Results

### Pulmonary and anthropometric characteristics

Characteristics of the study subjects are summarized in Table 1. COPD patients had moderate airflow obstruction (FEV_1_ 59±16). Compared to healthy controls, the COPD patients had a lower resting arterial oxygen saturation (*S*_p_,O_2_ 96 (95, 97) *vs* 98 (98, 100)%) and daily physical activity level (214 (129, 300) *vs* 328 (276, 489) counts/min). COPD_D_ patients did not differ from COPD_ND_ patients in terms of pulmonary function indices, but had a lower BMI.

**Table 1:** Main characteristics of subjects.

	Healthy controls	COPD	COPD_ND_	COPD_D_
N (M/F)	15 (9/6)	28 (16/12)	15 (9/6)	13 (7/6)
Age, y	65±6	65±7	66±7	65±6
Height, cm	170±11	169±9	168±7	169±11
Weight, kg	72±12	71±10	74±10	67±10
BMI, kg/m	24.9±3.3	25.1±2.8	26.4±2.5	23.5±2.3^#^
FFMI, kg/m	18.0±1.9	17.5±1.8	18.0±2.0	16.9±1.5
Smoking status current/former/never	1/7/7	10/18/0^**^	7/8/0^**^	3/10/0^*^
FEV_1_, % predicted	113±15	59±16^***^	61±13^***^	56±19^***^
FVC, % predicted	120±17	104±22^*^	98±20^*^	110±24
*D*_L,CO_, % predicted	95±19	51±16^***^	55±16^***^	47±16^***^
*S*_p_,o_2_ at rest, %	98 (98, 100)	96 (95, 97)^***^	97 (96, 97)^***^	95 (94, 98)^***^
*P*_a_,o_2_ at rest, kPa	12.1±1.1	9.3±1.0^***^	9.5±0.8^***^	9.1±1.2^***^
*P*_a_,co_2_ at rest, kPa	5.3±0.2	5.2±0.5	5.1±0.5	5.2±0.4
Daily physical activity, counts/min	328 (276, 489)	214 (129, 300)^**^	208 (140, 278)^**^	220 (108, 366)

Values are expressed as mean±SD or median (25th percentile, 75th percentile). Abbreviations: BMI, body mass index; FFMI, fat-free mass index; FEV_1_, forced expiratory volume in one second; FVC, forced vital capacity; *D*_L,CO_, diffusion capacity of the lungs for carbon monoxide; *S*p,o_2_, oxygen saturation measured via pulse oximetry. Significance of difference compared to controls: ^*^p<0.05, ^**^p<0.01, ^***^p≤0.001. Level of significance COPD_D_ *vs.* COPD_ND_ patients: ^#^p<0.05.

### Oxidative phenotype in quadriceps muscle

At baseline, as reported previously [8] and listed in Table 2, Oxphen markers were lower in COPD patients than in controls, which was reflected by lower type I and higher type II fiber proportions, and reduced content of some subunits of the oxidative phosphorylation complexes. Oxphen markers were not different between patients who did or did not desaturate during exercise (Table 2). Moreover, quadriceps endurance was reduced in this COPD cohort (decline in peak torque −1.74 (−2.04, −1.28) in COPD *vs* −1.09 (−1.57, −0.91)%/repetition in healthy controls), but it was not different between COPD_D_ and COPD_ND_ patients (−1.86 (−2.02, −1.35) in COPD_D_ *vs* −1.61 (−2.14, −1.25)%/repetition in COPD_ND_).

**Table 2:** Main characteristics of *vastus lateralis* muscle fibers.

	Healthy controls	COPD	COPD_ND_	COPD_D_
Type I proportion, %	62.7 (55.3, 68.3)	39.7 (35.3, 48.6)^***^	40.3 (35.5, 62.3)^*^	39.7 (29.8, 47.4)^***^
Type I/IIa proportion, %	8.1 (4.6, 11.0)	5.6 (2.2, 9.5)	5.3 (3.5, 9.0)	6.4 (1.2, 12.7)
Type IIa proportion, %	25.5 (16.8, 32.4)	40.5 (25.9, 48.9)^**^	32.0 (24.5, 46.1)^*^	45.4 (39.7, 55.2)^***^
Type IIa/IIx proportion, %	1.0 (0.3, 3.4)	3.6 (1.1, 8.1)	4.3 (1.0, 9.4)	3.6 (0.7, 7.4)
Type IIx proportion, %	0.5 (0.0, 2.7)	2.3 (0.5, 9.4)^*^	2.2 (0.2, 8.8)	3.2 (0.6, 11.0)
Type I fiber CSA, µm^2^	8703±1858	8154±2460	8524±2655	7756±2268
Type I/IIa fiber CSA, µm^2^	6540±2048	6073±2742	6274±2810	5838±2766
Type IIa fiber CSA, µm^2^	7107±2580	7016±2309	7030±2533	7001±2144
Type IIa/IIx fiber CSA, µm^2^	7214±3139	5760±2795	6082±3124	5341±2396
Type IIx fiber CSA, µm^2^	4694±2613	3792±2032	4375±2192	3257±1799
CS activity, µmol/min/g protein	63±37	54±22	52±24	56±21
HADH activity, µmol/min/g protein	22±9	19±7	19±7	19±6
OXPHOS complex I subunit content, AU	2.0 (1.2, 3.9)	1.5 (0.8, 2.1)	1.6 (1.0, 1.9)	1.2 (0.3, 2.4)
OXPHOS complex II subunit content, AU	2.8 (1.7, 4.3)	2.2 (1.5, 3.6)	2.3 (1.6, 2.5)	2.2 (1.2, 4.2)
OXPHOS complex III subunit content, AU	1.5 (0.7, 2.0)	1.2 (0.8, 1.7)	1.2 (0.9, 1.4)	1.4 (0.7, 1.8)
OXPHOS complex IV subunit content, AU	4.1 (2.8, 8.1)	2.4 (1.1, 3.6)^*^	2.9 (1.3, 4.1)	1.9 (0.6, 3.3)^*^
OXPHOS complex V subunit content, AU	0.7 (0.4, 1.1)	0.5 (0.3, 0.7)^*^	0.5 (0.3, 0.8)	0.6 (0.3, 0.7)

Values are expressed as mean±SD or median (25th percentile, 75th percentile). Abbreviations: CSA, cross-sectional area; CS, citrate synthase; HADH, 3-hydroxyacyl-CoA dehydrogenase; OXPHOS, oxidative phosphorylation; AU, arbitrary units. Significance of difference compared to controls: * p<0.05, ** p<0.01, *** p≤0.001.

### Cycle ergometry performance

Data from the cycle ergometry test are listed in Table 3. As expected, peak work rate was lower in COPD patients than in controls (72 (56, 96) *vs* 156 (134, 255) Watt). No differences were found between COPD_D_ and COPD_ND_ patients.

**Table 3:** Results from the maximal cycle ergometry test.

	Healthy controls	COPD	COPD_ND_	COPD_D_
Total exercise time, min	10.0 (8.8, 10.6)	7.4 (5.5, 9.4)^**^	7.5 (5.5, 8.9)^*^	7.0 (5.7, 9.7)^*^
Peak work load, Watt	156 (134, 255)	72 (56, 96)^***^	76 (66, 100)^***^	66 (53, 96)^***^
Work load, % predicted	133 (106, 152)	60 (44, 71)^***^	65 (48, 73)^***^	57 (37, 69)^***^
*V*′o_2peak_, ml/min	1852 (1680, 2785)	1130 (1025, 1406)^***^	1162 (1075, 1434)^***^	1084 (901, 1358)^***^
*V*′o_2peak_, % predicted	120 (102, 155)	74 (55, 84)^***^	78 (66, 88)^***^	64 (51, 74)^***^
*V*′e_,peak_, % MVV	66 (61, 80)	85 (81, 94)^***^	82 (77, 94)^**^	89 (82, 94)^**^
HR_peak_, % HR predicted	100 (97, 106)	78 (68, 89)^***^	77 (66, 89)^**^	78 (71, 92)^**^
AT*V*′o_2_, ml/min	1061 (862, 1395)	736 (607, 873)^***^	776 (601, 875)^**^	727 (605, 889)^**^
*S*_p_,o_2_ at *V*′o_2peak_, %	97 (97, 99)	92 (87, 95)^***^	94 (93, 96)^***^	87 (85, 90)^***, ###^
Δ*S*_p_,o_2_, %	−1.0 (−2.0, 0.0)	−4.0 (−8.0, −2.3)^***^	−3.0 (−4.0, 0.0)	−8.0 (−10.0, −6.5)^***, ###^
Δ*S*_a_,o_2_, %	0.0 (0.0, 1.0)	−1.0 (−4.0, 0.0)^*^	−1.0 (−1.8, 1.5)	−4.0 (−10.0, −3.0)^**, ##^

Values are expressed as median (25th percentile, 75th percentile). Abbreviations: *V*′o_2_, oxygen uptake; *V*′e, minute ventilation; MVV, maximal voluntary ventilation, calculated as FEV_1_×37.5; HR, heart rate; HR predicted calculated as (220 − age); AT*V*′o_2_, *V*′o_2_ at anaerobic threshold; *S*_p_,o_2_, oxygen saturation measured via pulse oximetry; *S*_a_,o_2_, arterial oxygen saturation. Level of significance of difference *vs.* controls: ^*^p<0.05, ^**^p<0.01, ^***^p≤0.001. Level of significance of difference COPD_D_ *vs.* COPD_ND_ patients: ^##^p<0.01, ^###^p≤0.001.

### Exercise-induced metabolic gene expression

**Figure 1:**
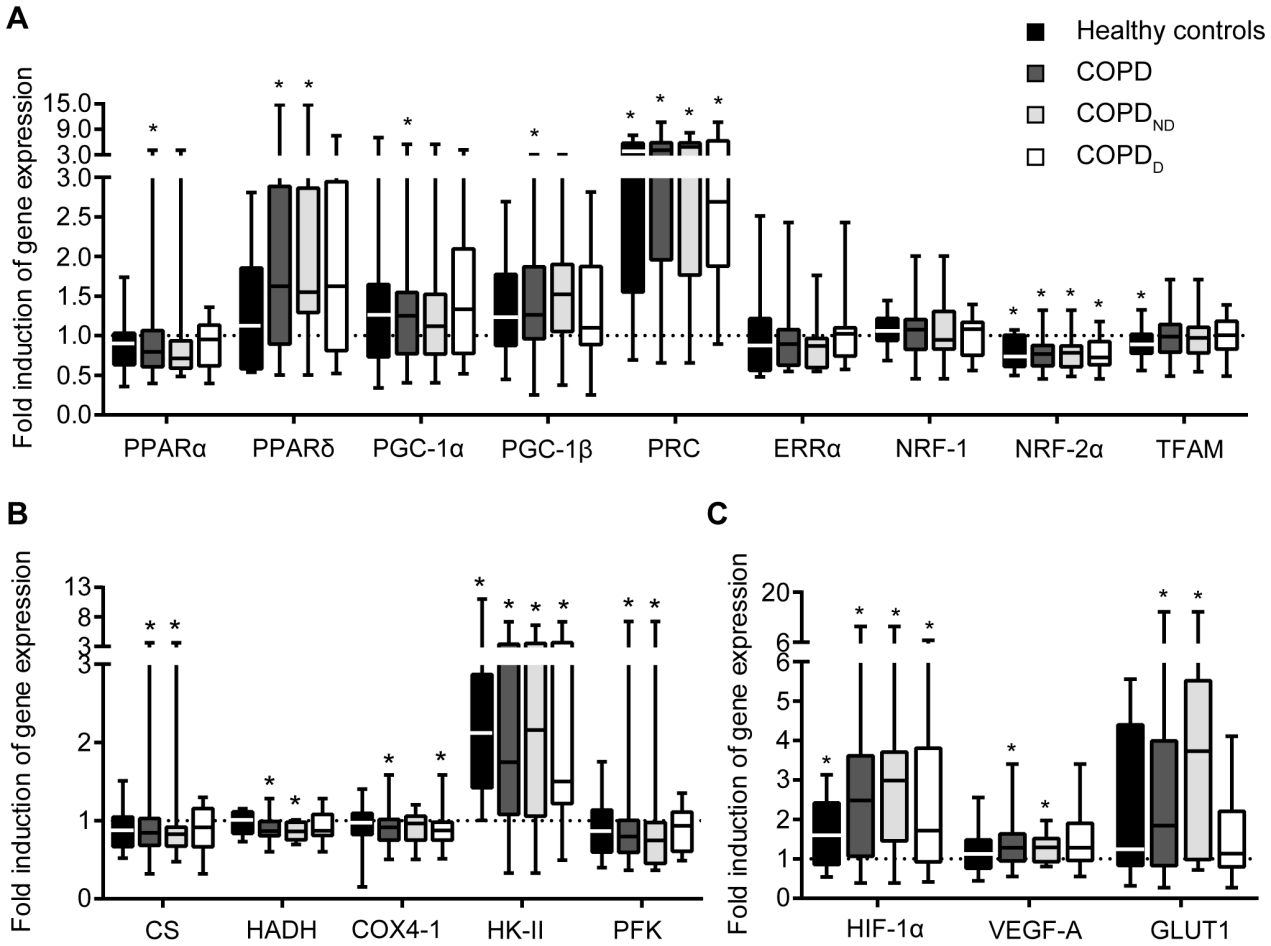
Gene expression responses to exercise are not significantly different between COPD patients and healthy controls, regardless of desaturation status. **A** Gene expression inductions (post/pre) for regulators of Oxphen. PPAR, peroxisome proliferator-activated receptor; PGC, PPAR gamma co-activator; PRC, PGC-1-related co-activator; ERRα, estrogen-related receptor α; NRF, nuclear respiratory factor; TFAM, mitochondrial transcription factor A. **B** Gene expression inductions for metabolic enzymes involved in citric acid cycle, β-oxidation and glycolysis. CS, citrate synthase; HADH, 3-hydroxyacyl-CoA dehydrogenase; COX4-1, cytochrome *c* oxidase subunit IV isoform 1; HK-II, hexokinase II; PFK, phosphofructokinase muscle isoform. **C** Gene expression inductions of hypoxia-associated targets. HIF-1α, hypoxia-inducible factor 1 alpha; VEGF-A, vascular endothelial growth factor A; GLUT1, glucose transporter 1. Whiskers indicate minimum and maximum values. Level of significance: ^*^p<0.05 for post *vs*. pre.

The acute exercise clearly evoked an upregulation of genes known to be exercise-responsive, including PGC-1α-related co-activator PRC, glycolysis-involved hexokinase II and the master regulator of the hypoxic response, HIF-1α (Figure 1). The COPD patients did not respond differently to exercise than controls regarding expression of genes associated with muscle oxidative metabolism, nor of genes involved in glycolytic metabolism or hypoxia signaling. We also did not observe a different response in COPD_D_ patients compared to COPD_ND_ patients.

### Exercise response of oxidative stress markers

**Figure 2:**
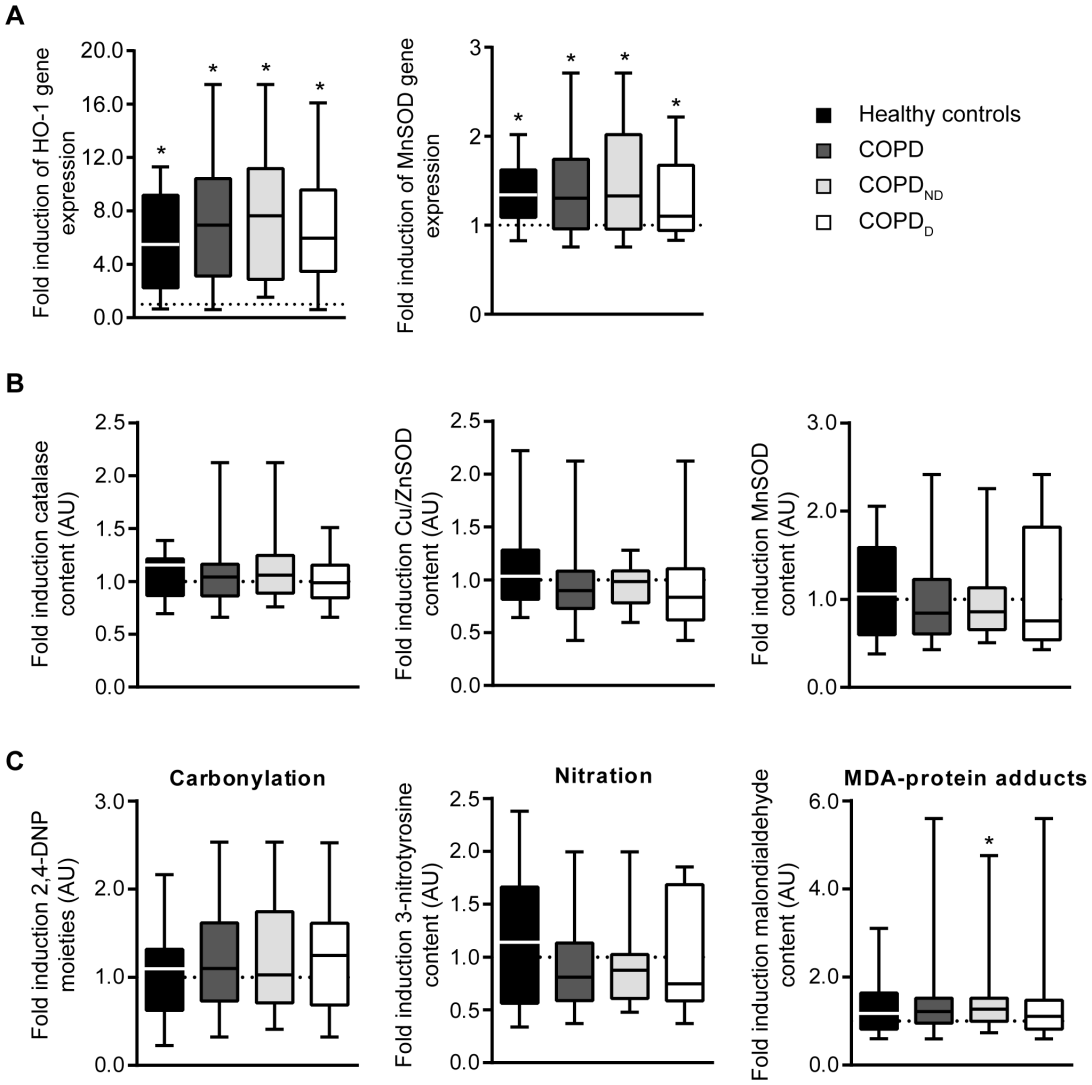
Oxidative stress in quadriceps muscle is only mildly increased in non-desaturating COPD patients; both patients and healthy controls show significant induction of antioxidant genes after exercise. **A** Box plots of the response of heme oxygenase-1 (HO-1) and manganese superoxide dismutase (MnSOD) genes to exercise presented as fold induction (post/pre). **B** Box plot of the exercise-induced fold inductions of catalase, Cu/ZnSOD and MnSOD content. **C** Box plot of the exercise-induced fold inductions of protein carbonylation, tyrosine nitration and malondialdehyde (MDA)-protein adducts. Whiskers indicate minimum and maximum values. AU, arbitrary units. Level of significance: ^*^p<0.05 for post *vs*. pre.

At baseline, oxidative stress-induced protein modification levels such as carbonylation, tyrosine nitration and MDA adducts were not different between COPD patients and controls [8], nor between COPD_D_ and COPD_ND_ patients (data not shown). After exercise, gene expression levels of heme oxygenase-1 (HO-1) and manganese superoxide dismutase (MnSOD) were increased in both COPD patients and healthy controls (Figure 2A). An exercise response of the antioxidant enzymes was not detected at the protein level as manganese and copper/zinc superoxide dismutase and catalase protein content remained unchanged (Figure 2B). No differential response was shown between COPD_D_ and COPD_ND_ patients.

The increases in HO-1 and MnSOD gene expression did not correlate with exercise-induced oxidative stress-derived products; only MDA-protein adducts were slightly increased (Figure 2C) in COPD_ND_ patients.

### Leg muscle desaturation

**Figure 3:**
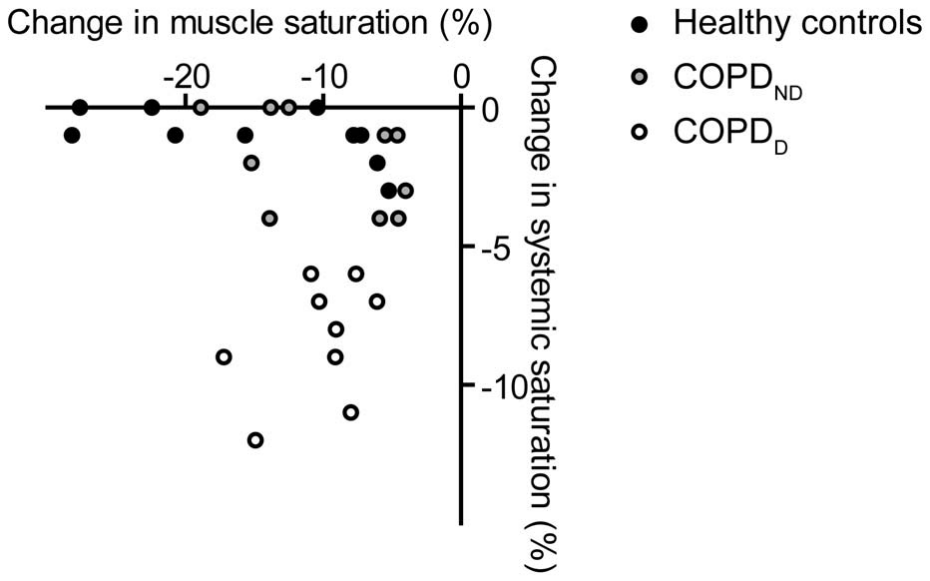
Change in quadriceps muscle saturation during exercise is not associated with systemic desaturation. Reliable muscle saturation data was available for 11 COPD_ND_, 9 COPD_D_ patients and 11 healthy controls.

Although by definition COPD_D_ patients had significantly reduced end-exercise systemic saturation compared to COPD_ND_ patients and controls, no differences were detected in quadriceps tissue desaturation or time constant of recovery of muscle oxygen saturation among the groups (Table 4). Also, no association was found between systemic and local muscle desaturation (Figure 3). To assess whether the Oxphen response was influenced by muscle oxygen saturation, we tested correlations between Oxphen markers and ΔTSI in the combined group of COPD patients and controls, but did not find significant associations. In addition, we stratified the COPD patients based on the median value of ΔTSI to high (≤−9.1%) and low muscle desaturation (>−9.1%) groups, due to lack of a reference value for muscle desaturation. The response of Oxphen markers was not different between the two groups.

**Table 4:** Quadriceps desaturation during cycle ergometry.

	Healthy controls	COPD	COPD_ND_	COPD_D_
ΔTSI, %	−13 (−24, −7)	−9 (−14, −6)	−6 (−14, −5)	−9 (−13, −8)
*τ*, s	24 (17, 26)	25 (18, 40)	28 (18, 46)	24 (18, 37)

Values are expressed as median (25th percentile, 75th percentile). Abbreviations: TSI, tissue saturation index; *τ*, time constant of recovery of muscle oxygen saturation.

## Discussion

The mechanisms leading to loss of muscle Oxphen in COPD thus far remain unclear. We recently showed that the Oxphen loss is not limited to advanced disease but also ongoing in earlier phases of the disease, even in absence of muscle wasting. Potential determinants such as disease-associated persistent systemic inflammation and chronic hypoxia are less likely to be causally involved in this stage. As exercise is a potent Oxphen stimulant we hypothesized that a blunted regulatory Oxphen response to exercise would occur in less advanced COPD, resulting from either exercise-induced hypoxia or oxidative stress, or both. However, we did not demonstrate such a blunted response in quadriceps muscle, regardless of co-occurring oxygen desaturation. Also exercise-induced oxidative stress was not aggravated in these patients with less advanced COPD compared to controls. Moreover, as the COPD patients exercised at an intensity significantly lower than that of healthy controls, it could be argued that, adjusted for absolute intensity, the exercise response was even larger in the patients as compared to the controls. In line, after a constant load test at 80% peak capacity in COPD patients and healthy controls, Steiner *et al.* also did not find significantly different exercise-induced changes in absolute magnitude of the muscle metabolites ATP, inosine monophosphate, phosphocreatine and lactate, despite marked differences in workload between COPD patients and controls [36].

### Exercise intensity and response

One explanation for the comparable exercise responses in the COPD patients and healthy controls could be that the induction of Oxphen is not linearly related to exercise intensity but rather determined by a threshold that has to be exceeded to invoke a maximal response. The fact that in our study the anaerobic threshold was reached in all healthy controls and almost all COPD patients would be supportive of this hypothesis. Congruently, in healthy individuals only exercise above the lactate threshold has been shown to significantly induce muscle PGC-1α gene expression [37], [38]. In COPD patients, however, exercise both below and above the lactate threshold increased PGC-1α gene expression, although the induction was stronger with the latter [38]. Puente-Maestu *et al.* found increased induction of PGC-1α mRNA in muscle of COPD patients compared to healthy controls after a 45-minutes exercise test at 65% of *V*′o_2peak_ [38]. Whereas the COPD patients in our study had significantly increased PGC-1α gene expression after exercise compared to baseline, the induction in healthy controls only showed a trend to significance. However, we did not observe a significant difference in the magnitude of the exercise-associated induction between the patients and controls. Puente-Maestu *et al.* found an association of PGC-1α mRNA induction with the amount of ROS that was estimated to be produced during the exercise test [38]. In our study there was no evidence of increased oxidative stress in the quadriceps muscle of COPD patients, which together with the lack of a differential response of PGC-1α mRNA with exercise, is in line with the findings by Puente-Maestu.

### Regulatory gene responses to exercise

Transient induction of PGC-1α gene expression by exercise is well-described (i.e. [13]). Remarkably, in addition to a mild induction of PGC-1α gene expression, we found a strong induction of PRC, a PGC-1-related coactivator involved in mitochondrial respiratory function. Induction of PRC with exercise has been described before in healthy individuals [39], but this is to our knowledge the first time that this marker has been measured in COPD patients in response to acute exercise.

Exercise-induced elevation of HIF-1α gene expression has been proposed to be an important factor in the regulation of adaptive gene responses to exercise [40], [41]. In addition to HIF-1α, we found increased expression of some of its target genes, such as VEGF-A, HK-II and GLUT1. The inductions of these genes and PGC-1α and PRC support that there is an ongoing exercise response in the muscle. It is thought that induction of the HIF-1α gene with exercise does not depend on hypoxia *per se*, but may result in increased capillarization and increased glycolytic flux within the mitochondria [40]. Inductions of HIF-1α gene expression have been found to blunt with training [40], and the seemingly stronger inductions of HIF-1α gene expression and its target genes in COPD patients compared to controls could be a reflection of the on average lower physical activity level of the patients.

### Exercise-associated hypoxia

In contrast to what we expected, exercise-induced desaturation did not blunt the Oxphen response in COPD patients. This could indicate that acute local hypoxia does not hamper the Oxphen response. Another explanation for the comparable exercise responses between the COPD_ND_ and COPD_D_ subgroups is that “systemic” oxygen desaturation as measured by finger pulse oximetry does not reflect oxygen desaturation locally in the exercising limb muscle. To define exercise-induced oxygen desaturation, we used the criterion by the American Thoracic Society (ATS), which specifies that the drop in saturation during exercise should be ≥4% [26]. It has also been suggested that desaturation is only clinically relevant when saturation drops below 90% [42], which applied to 12 patients in the current study. However, the outcomes did not change when we used this alternative criterion (results not shown). Indeed, we did not find an association between systemic and skeletal muscle desaturation. The mismatch between systemic and muscle desaturation could be explained by an increased oxygen extraction rate from the systemic circulation to maintain oxygen tension in the muscle but resulting in decreased arterial oxygen saturation in some patients. Nevertheless, we found no significant difference in exercise-induced muscle oxygen desaturation levels among the groups, including the healthy controls, and no associations of muscle desaturation with markers of the Oxphen response.

### Exercise-associated oxidative stress response

Previous research in advanced COPD demonstrated an increased resting and exercise-induced oxidative stress response. We also investigated relevant oxidative stress markers in this study to see if a similar enhancement is seen in less advanced COPD, in particular in desaturating patients. No striking differences were observed between COPD patients, subgroups and healthy controls. In contrast Puente-Maestu *et al.* recently described increased exercise-induced oxidative stress in mild to moderate COPD patients, who, similar to those in our study, had a slight loss of muscle Oxphen [43]. However, differences in the intensity of exercise (15 minutes constant work rate at ∼65% W_max_ *vs* incremental exercise until exhaustion) and the timing of the muscle biopsy after the exercise test (1 h *vs* 4 h) hamper comparison between our study and theirs.

Although we did not find increased oxidative stress-derived products after exercise, gene expression levels of the antioxidants MnSOD and HO-1 were increased, which is a normal response to exercise [28], [44]. The induction of MnSOD and HO-1 gene expression could indicate that the stimulation of the antioxidant system by exercise is not blunted in COPD patients and increased formation of reactive oxygen species by exercise is well-balanced by the antioxidant system [45].

### Limitations of the study

A limitation of this study is that only one biopsy was taken after the exercise bout. The timing of the single post biopsy was chosen after careful review of the literature focusing on Oxphen gene expression after exercise, but no inferences can be made regarding differences in the temporal pattern of gene expression changes between COPD patients and controls.

An important constraint of the near-infrared spectroscopy (NIRS) technique is the limited optical penetration depth. As such, subcutaneous fat disturbs or even obstructs the quadriceps NIRS measurement. Therefore, subjects with a large skin fold at the site of the NIRS probe were excluded from the NIRS measurement in our study. We explicitly report this methodological issue as it often seems to be overlooked in the existing literature.

## Conclusion

Taken together, the data in this study suggest that patients with less advanced COPD do not have a blunted muscle oxidative regulatory response to acute exercise, regardless of exercise-induced systemic desaturation. This observation, together with a normal oxidative stress response, further supports that incorporation of exercise training as integrated part of COPD management already in early disease could have a pivotal role to prevent or slow down loss of muscle Oxphen and related functional impairment. Further research is needed to investigate if the muscle oxidative regulatory response is blunted in more severe disease stages (i.e. chronic respiratory failure) and disentangle potential contributing factors that may require additional modulation to optimize exercise training in these stages.

## Supporting Information

File S1 Table S1. qRT-PCR primer details for genes of interest.Table S2. qRT-PCR primer details for reference genes.
(DOCX)

## References

[1] Gosker HR, Zeegers MP, Wouters EF, Schols AM (2007) Muscle fibre type shifting in the vastus lateralis of patients with COPD is associated with disease severity: a systematic review and meta-analysis. Thorax 62: 944–949. 17526675 10.1136/thx.2007.078980 PMC2117111

[2] Maltais F, LeBlanc P, Whittom F, Simard C, Marquis K, et al (2000) Oxidative enzyme activities of the vastus lateralis muscle and the functional status in patients with COPD. Thorax 55: 848–853. 10992537 10.1136/thorax.55.10.848 PMC1745616

[3] Maltais F, Sullivan MJ, LeBlanc P, Duscha BD, Schachat FH, et al (1999) Altered expression of myosin heavy chain in the vastus lateralis muscle in patients with COPD. Eur Respir J 13: 850–854. 10362052 10.1034/j.1399-3003.1999.13d26.x

[4] Allaire J, Maltais F, Doyon JF, Noel M, LeBlanc P, et al (2004) Peripheral muscle endurance and the oxidative profile of the quadriceps in patients with COPD. Thorax 59: 673–678. 15282387 10.1136/thx.2003.020636 PMC1747097

[5] Layec G, Haseler LJ, Hoff J, Richardson RS (2011) Evidence that a higher ATP cost of muscular contraction contributes to the lower mechanical efficiency associated with COPD: preliminary findings. Am J Physiol Regul Integr Comp Physiol 300: R1142–1147. 21307358 10.1152/ajpregu.00835.2010 PMC3293514

[6] van den Borst B, Gosker HR, Schols AM (2013) Central fat and peripheral muscle: partners in crime in chronic obstructive pulmonary disease. Am J Respir Crit Care Med 187: 8–13. 23281350 10.1164/rccm.201208-1441OE

[7] Remels AH, Gosker HR, Langen RC, Schols AM (2013) The mechanisms of cachexia underlying muscle dysfunction in COPD. J Appl Physiol (1985) 114: 1253–1262. 23019314 10.1152/japplphysiol.00790.2012

[8] van den Borst B, Slot IG, Hellwig VA, Vosse BA, Kelders MC, et al (2013) Loss of quadriceps muscle oxidative phenotype and decreased endurance in patients with mild-to-moderate COPD. J Appl Physiol (1985) 114: 1319–1328. 22815389 10.1152/japplphysiol.00508.2012

[9] Park SK, Richardson CR, Holleman RG, Larson JL (2013) Physical activity in people with COPD, using the National Health and Nutrition Evaluation Survey dataset (2003-2006). Heart Lung 42: 235–240. 23726356 10.1016/j.hrtlng.2013.04.005 PMC4031646

[10] Gouzi F, Maury J, Molinari N, Pomies P, Mercier J, et al (2013) Reference values for vastus lateralis fiber size and type in healthy subjects over 40 years old: a systematic review and metaanalysis. J Appl Physiol (1985) 115: 346–354. 23558383 10.1152/japplphysiol.01352.2012

[11] Natanek SA, Gosker HR, Slot IG, Marsh GS, Hopkinson NS, et al (2013) Heterogeneity of quadriceps muscle phenotype in chronic obstructive pulmonary disease (Copd); implications for stratified medicine? Muscle Nerve 48: 488–497. 23553751 10.1002/mus.23784

[12] Mahoney DJ, Parise G, Melov S, Safdar A, Tarnopolsky MA (2005) Analysis of global mRNA expression in human skeletal muscle during recovery from endurance exercise. FASEB J 19: 1498–1500. 15985525 10.1096/fj.04-3149fje

[13] Pilegaard H, Saltin B, Neufer PD (2003) Exercise induces transient transcriptional activation of the PGC-1alpha gene in human skeletal muscle. J Physiol 546: 851–858. 12563009 10.1113/jphysiol.2002.034850 PMC2342594

[14] Itoh K, Itoh M, Ishihara A, Hirofuji C, Hayashi H (1995) Influence of 12 weeks of hypobaric hypoxia on fibre type composition of the rat soleus muscle. Acta Physiol Scand 154: 417–418. 7572239 10.1111/j.1748-1716.1995.tb09925.x

[15] Mason SD, Howlett RA, Kim MJ, Olfert IM, Hogan MC, et al (2004) Loss of skeletal muscle HIF-1alpha results in altered exercise endurance. PLoS Biol 2: e288. 15328538 10.1371/journal.pbio.0020288 PMC514537

[16] Hoppeler H, Vogt M, Weibel ER, Fluck M (2003) Response of skeletal muscle mitochondria to hypoxia. Exp Physiol 88: 109–119. 12525860 10.1113/eph8802513

[17] Magalhaes J, Ascensao A, Soares JM, Ferreira R, Neuparth MJ, et al (2005) Acute and severe hypobaric hypoxia increases oxidative stress and impairs mitochondrial function in mouse skeletal muscle. J Appl Physiol (1985) 99: 1247–1253. 15905323 10.1152/japplphysiol.01324.2004

[18] Koechlin C, Maltais F, Saey D, Michaud A, LeBlanc P, et al (2005) Hypoxaemia enhances peripheral muscle oxidative stress in chronic obstructive pulmonary disease. Thorax 60: 834–841. 15964914 10.1136/thx.2004.037531 PMC1747208

[19] Mercken EM, Gosker HR, Rutten EP, Wouters EF, Bast A, et al (2009) Systemic and pulmonary oxidative stress after single-leg exercise in COPD. Chest 136: 1291–1300. 19696125 10.1378/chest.08-2767

[20] Barreiro E, Rabinovich R, Marin-Corral J, Barbera JA, Gea J, et al (2009) Chronic endurance exercise induces quadriceps nitrosative stress in patients with severe COPD. Thorax 64: 13–19. 18835959 10.1136/thx.2008.105163

[21] Couillard A, Maltais F, Saey D, Debigare R, Michaud A, et al (2003) Exercise-induced quadriceps oxidative stress and peripheral muscle dysfunction in patients with chronic obstructive pulmonary disease. Am J Respir Crit Care Med 167: 1664–1669. 12672647 10.1164/rccm.200209-1028OC

[22] Picard M, Godin R, Sinnreich M, Baril J, Bourbeau J, et al (2008) The mitochondrial phenotype of peripheral muscle in chronic obstructive pulmonary disease: disuse or dysfunction? Am J Respir Crit Care Med 178: 1040–1047. 18755922 10.1164/rccm.200807-1005OC

[23] Anderson EJ, Neufer PD (2006) Type II skeletal myofibers possess unique properties that potentiate mitochondrial H(2)O(2) generation. Am J Physiol Cell Physiol 290: C844–851. 16251473 10.1152/ajpcell.00402.2005

[24] Franssen FM, Broekhuizen R, Janssen PP, Wouters EF, Schols AM (2005) Limb muscle dysfunction in COPD: effects of muscle wasting and exercise training. Med Sci Sports Exerc 37: 2–9. 15632660 10.1249/01.mss.0000150082.59155.4f

[25] Beaver WL, Wasserman K, Whipp BJ (1986) A new method for detecting anaerobic threshold by gas exchange. J Appl Physiol (1985) 60: 2020–2027. 3087938 10.1152/jappl.1986.60.6.2020

[26] American Thoracic S (2003) American College of Chest P (2003) ATS/ACCP Statement on cardiopulmonary exercise testing. Am J Respir Crit Care Med 167: 211–277. 12524257 10.1164/rccm.167.2.211

[27] Bergström J (1962) Muscle electrolytes in man. Determination by neutron activation analysis on needle biopsy specimens. A study on normal subjects, kidney patients, and patients with chronic diarrhea. Scand J Clin Lab Invest 14: 1–110.

[28] Pilegaard H, Ordway GA, Saltin B, Neufer PD (2000) Transcriptional regulation of gene expression in human skeletal muscle during recovery from exercise. Am J Physiol Endocrinol Metab 279: E806–814. 11001762 10.1152/ajpendo.2000.279.4.E806

[29] Yang Y, Creer A, Jemiolo B, Trappe S (2005) Time course of myogenic and metabolic gene expression in response to acute exercise in human skeletal muscle. J Appl Physiol (1985) 98: 1745–1752. 15618316 10.1152/japplphysiol.01185.2004

[30] Ogilvie RW, Feeback DL (1990) A metachromatic dye-ATPase method for the simultaneous identification of skeletal muscle fiber types I, IIA, IIB and IIC. Stain Technol 65: 231–241. 1703671 10.3109/10520299009105613

[31] Allen D, Winters E, Kenna PF, Humphries P, Farrar GJ (2008) Reference gene selection for real-time rtPCR in human epidermal keratinocytes. J Dermatol Sci 49: 217–225. 18061409 10.1016/j.jdermsci.2007.10.001

[32] Vandesompele J, De Preter K, Pattyn F, Poppe B, Van Roy N, et al (2002) Accurate normalization of real-time quantitative RT-PCR data by geometric averaging of multiple internal control genes. Genome Biol 3: RESEARCH0034. 12184808 10.1186/gb-2002-3-7-research0034 PMC126239

[33] Bergmeyer HU, Gawehn K, Grassl M (1974) 3-Hydroxyacyl-CoA dehydrogenase. In: Bergmeyer HU, editor. Methods of enzymatic analysis. Weinheim; Germany: Verlag Chemie GmbH. pp. 474.

[34] Ling KH, Paetkau V, Marcus F, Lardy HA (1966) Phosphofructokinase: I. Skeletal Muscle. Methods Enzymol: Academic Press. pp. 425-429.

[35] Shepherd D, Garland PB (1969) Citrate synthase from rat liver. Methods Enzymol: Academic Press. pp. 11-16.

[36] Steiner MC, Evans R, Deacon SJ, Singh SJ, Patel P, et al (2005) Adenine nucleotide loss in the skeletal muscles during exercise in chronic obstructive pulmonary disease. Thorax 60: 932–936. 16055624 10.1136/thx.2004.038802 PMC1747228

[37] Tobina T, Yoshioka K, Hirata A, Mori S, Kiyonaga A, et al (2011) Peroxisomal proliferator-activated receptor gamma co-activator-1 alpha gene expression increases above the lactate threshold in human skeletal muscle. J Sports Med Phys Fitness 51: 683–688. 22212273

[38] Puente-Maestu L, Lazaro A, Tejedor A, Camano S, Fuentes M, et al (2011) Effects of exercise on mitochondrial DNA content in skeletal muscle of patients with COPD. Thorax 66: 121–127. 21097816 10.1136/thx.2010.153031

[39] Russell AP, Hesselink MK, Lo SK, Schrauwen P (2005) Regulation of metabolic transcriptional co-activators and transcription factors with acute exercise. FASEB J 19: 986–988. 15814608 10.1096/fj.04-3168fje

[40] Lundby C, Gassmann M, Pilegaard H (2006) Regular endurance training reduces the exercise induced HIF-1alpha and HIF-2alpha mRNA expression in human skeletal muscle in normoxic conditions. Eur J Appl Physiol 96: 363–369. 16284786 10.1007/s00421-005-0085-5

[41] Ameln H, Gustafsson T, Sundberg CJ, Okamoto K, Jansson E, et al (2005) Physiological activation of hypoxia inducible factor-1 in human skeletal muscle. FASEB J 19: 1009–1011. 15811877 10.1096/fj.04-2304fje

[42] Stoller JK, Panos RJ, Krachman S, Doherty DE, Make B, et al (2010) Oxygen therapy for patients with COPD: current evidence and the long-term oxygen treatment trial. Chest 138: 179–187. 20605816 10.1378/chest.09-2555 PMC2897694

[43] Puente-Maestu L, Tejedor A, Lazaro A, de Miguel J, Alvarez-Sala L, et al (2012) Site of mitochondrial reactive oxygen species production in skeletal muscle of chronic obstructive pulmonary disease and its relationship with exercise oxidative stress. Am J Respir Cell Mol Biol 47: 358–362. 22493009 10.1165/rcmb.2011-0382OC

[44] Hollander J, Fiebig R, Gore M, Ookawara T, Ohno H, et al (2001) Superoxide dismutase gene expression is activated by a single bout of exercise in rat skeletal muscle. Pflugers Arch 442: 426–434. 11484775 10.1007/s004240100539

[45] Gosker HR, Bast A, Haenen GR, Fischer MA, van der Vusse GJ, et al (2005) Altered antioxidant status in peripheral skeletal muscle of patients with COPD. Respir Med 99: 118–125. 15672860 10.1016/j.rmed.2004.05.018

