# Supplementary material for: The Muscle Oxidative Regulatory Response to Acute Exercise Is Not Impaired in Less Advanced COPD Despite a Decreased Oxidative Phenotype
Source: PLoS One. 2014 Feb 28;9(2):e90150. doi: 10.1371/journal.pone.0090150 (PMC3938598; doi:10.1371/journal.pone.0090150)
Supplement: File S1 — Table S1. qRT-PCR primer details for genes of interest.Table S2. qRT-PCR primer details for reference genes. (DOCX) [file pone.0090150.s001.docx]

# The muscle oxidative regulatory response to acute exercise is not impaired in less advanced COPD despite a decreased oxidative phenotype

Ilse G.M. Slot, Bram van den Borst, Valéry A.C.V. Hellwig, Esther Barreiro, Annemie M.W.J. Schols, Harry R. Gosker

# Online supplement

### Table S1. qRT‑PCR primer details for genes of interest

| Symbol | Name | Ensembl ID | Forward primer (5'⭢3') | Reverse primer (5'⭢3') | Amplicon size (bp) |
| --- | --- | --- | --- | --- | --- |
| *COX4I1* | Cytochrome c oxidase subunit IV isoform 1 | ENSG00000131143 | CCATGGATGAGAAAGTCGAGT | CGTTCGAGCCCCTGTTCA | 75 |
| *CS* | Citrate synthase | ENSG00000062485 | GATGTGTCAGATGAGAAGTTACGAGACT | TGGCCATAGCCTGGAACAA | 77 |
| *ESRRA* | Estrogen-related receptor | ENSG00000173153 | GCGGCTGGAGCGAGAGGAG | CTCGGCATCTTCGATGTGCAC | 82 |
| *GABPA* | GA binding protein alpha; nuclear respiratory factor 2 alpha | ENSG00000154727 | CTCACCTGGGAACAGAACAGGAA | ACCCAAGAAATGCAGTCTCGAGC | 102 |
| *HADHA* | 3‑hydroxyacyl-CoA dehydrogenase/3‑ketoacyl-CoA thiolase/enoyl-CoA hydratase alpha | ENSG00000084754 | TGGCTTCCCGCCTTGTC | TGGAGCCGGTCCACTATCTTC | 78 |
| *HIF1A* | Hypoxia inducible factor 1 alpha | ENSG00000100644 | TGAACATAAAGTCTGCAACATGGA | TGAGGTTGGTTACTGTTGGTATCATATA | 82 |
| *HK2* | Hexokinase II | ENSG00000159399 | GTAAATACAGTGGATCTCAATCTTCGGG | CAAGGATTTGAGATGATTCGCTATTCA | 61 |
| *HMOX1* | Heme oxygenase-1 | ENSG00000100292 | CACCCAGGCAGAGAATGCTGAGTTC | GCCGCTTCACATAGCGCTGCA | 278 |
| *NRF1* | Nuclear respiratory factor 1 | ENSG00000106459 | ACGTGGCCAGACACAGTCACC | TGGTCACCTCCGCCTGAGTTT | 83 |
| *PFKM* | PFK muscle | ENSG00000152556 | CCTGCCCCTCATGGAATGT | GGGCTTCGTCAAATTTCTTCTC | 77 |
| *PPARGC1A* | Peroxisome proliferator-activated receptor gamma coactivator 1 alpha | ENSG00000109819 | GACCAGTGCTACCTGAGAGAGACTT | GCTCGGCTCGGATTTCCT | 97 |
| *PPARGC1B* | Peroxisome proliferator-activated receptor gamma coactivator 1 beta | ENSG00000155846 | GGCGCTTTGAAGTGTTTGGTG | TGATGAAGCCGTACTTCTCGCCT | 81 |
| *PPARA* | Peroxisome proliferator-activated receptor alpha | ENSG00000186951 | CAGAACAAGGAGGCGGAGGTC | AGGTCCAAGTTTGCGAAGC | 116 |
| *PPARD* | Peroxisome proliferator-activated receptor delta | ENSG00000112033 | TGACCAAAAAGAAGGCCCGC | GTCGTGGATCACAAAGGGCG | 71 |
| *PPRC1* | Peroxisome proliferator-activated receptor gamma coactivator-related 1 | ENSG00000148840 | GCCCTTTGATCTCTGCTTTGGG | AAGTCTTCCCGGTTGGAGTCAAG | 81 |
| *SLC2A1* | Glucose transporter (GLUT) 1 | ENSG00000117394 | TCTGGGCTGCCGGGTTCTAG | TTTGCAGGCTCCCACAGGC | 81 |
| *SOD2* | Mitochondrial superoxide dismutase 2; MnSOD | ENSG00000112096 | TGGACAAACCTCAGCCCTAACG | TGATGGCTTCCAGCAACTCCC | 60 |
| *TFAM* | Mitochondrial transcription factor A | ENSG00000108064 | GAAAGATTCCAAGAAGCTAAGGGTGATT | TCCAGTTTTCCTTTACAGTCTTCAGCTTTT | 67 |
| *VEGFA* | Vascular endothelial growth factor A | ENSG00000112715 | CCAGGCCCTCGTCATTG | AAGGAGGAGGGCAGAATCAT | 186 |

### Table S2. qRT‑PCR primer details for reference genes

| Symbol | Name | Ensembl ID | Primers (5'⭢3') | Reverse primer (5'⭢3') | Amplicon size (bp) |
| --- | --- | --- | --- | --- | --- |
| *ALAS1* | 5-Aminolevulinate synthase 1 | ENSG00000023330 | CTGCAAAGATCTGACCCCTC | CCTCATCCACGAAGGTGATT | 136 |
| *ACTB* | Beta-actin | ENSG00000075624 | AAGCCACCCCACTTCTCTCTAA | AATGCTATCACCTCCCCTGTGT | 73 |
| *B2M* | Beta-2-microglobulin | ENSG00000166710 | CTGTGCTCGCGCTACTCTCTCTT | TGAGTAAACCTGAATCTTTGGAGTACGC | 71 |
| *PPIA* | Peptidyl-prolyl cis-trans isomerase A; Cyclophilin A | ENSG00000196262 | CATCTGCACTGCCAAGACTGA | TTCATGCCTTCTTTCACTTTGC | 72 |
| *GAPDH* | Glyceraldehyde-3-phosphate dehydrogenase | ENST00000229239 | GCACCACCAACTGCTTAGCA | TGGCAGTGATGGCATGGA | 96 |
| *GUSB* | Beta-glucuronidase | ENSG00000169919 | CTCATTTGGAATTTTGCCGATT | CCGAGTGAAGATCCCCTTTTTA | 81 |
| *HMBS* | Hydroxymethyl-bilane synthase | ENSG00000256269 | GGCAATGCGGCTGCAA | GGGTACCCACGCGAATCAC | 64 |
| *HPRT1* | Hypoxanthine phosphoribosyl-transferase 1 | ENSG00000165704 | TGACACTGGCAAAACAATGCA | GGTCCTTTTCACCAGCAAGCT | 94 |
| *RPL13A* | Ribosomal protein 13A | ENSG00000142541 | CCTGGAGGAGAAGAGGAAAGAGA | TTGAGGACCTCTGTGTATTTGTCAA | 126 |
| *RPLP0* | Large ribosomal protein P0 | ENSG00000089157 | TCTACAACCCTGAAGTGCTTGATATC | GCAGACAGACACTGGCAACATT | 90 |
| *UBC* | Ubiquitin C | ENSG00000150991 | ATTTGGGTCGCAGTTCTTG | TGCCTTGACATTCTCGATGGT | 133 |
| *YWHAZ* | Tyrosine 3-monooxygenase/ tryptophan 5-mono-oxygenase activation protein, zeta polypeptide | ENSG00000164924 | ACTTTTGGTACATTGTGGCTTCAA | CCGCCAGGACAAACCAGTAT | 94 |
